# Bulbus Destruction by Choroidal Melanocytoma in a Dog: A 3-Year History

**DOI:** 10.3390/vetsci9060267

**Published:** 2022-06-01

**Authors:** Nadine Nautscher, Martin Steffl, Katharina Schmon, Eva Ludwig

**Affiliations:** 1 Institute of Animal Science, Veterinary Practice of the University of Hohenheim, 70599 Stuttgart, Germany; martin.steffl@uni-hohenheim.de (M.S.); katharina.schmon@uni-hohenheim.de (K.S.); 2 Specialized Practice for Veterinary Pathology, 80689 Munich, Germany; el@tierpathologie-muenchen.de

**Keywords:** choroidal melanocytoma, dog, uveitis, retinal detachment, secondary glaucoma, enucleation

## Abstract

A 3-year-old male Slovak Hound with retinal detachment was presented. The causative intraocular mass was detected by ultrasonography, and the course of the disease was monitored over a 3-year period. Enucleation was performed due to secondary glaucoma. A benign choroidal melanocytoma was diagnosed by histopathology. To our knowledge, this is the first report that describes the disease over such a long period of time. The mild course of the disease questions enucleation of eyes with no or minor symptoms. Conventional treatment may be a suitable alternative to surgery for dogs with high anesthesia risks.

## 1. Introduction

Uveal melanocytic neoplasms are the most common primary intraocular tumors in dogs [1]. Melanocytic neoplasms are described as 73–77% benign and 23–27% malignant [1,2,3], with an average occurrence at the age of 9 years [3]. No breed or sex predisposition have been reported [2]. Melanocytomas mostly arise from the anterior uveal tract, and only 6% affect the choroid [1]. The posterior location of the choroidal melanocytoma is difficult to diagnose until the tumor causes ocular diseases. Tumor infiltration into the surrounding tissue leads to chronic uveitis, secondary glaucoma, retinal detachment and intraocular hemorrhage [4]. The prognosis for dogs with choroidal melanocytoma is considered good [1], whereas the survival time for dogs with malignant melanomas is significantly shortened [3]. The malignant melanomas do not tend to metastasize [2,5], although occasional reports about metastases exist [6,7]. It is not possible to differentiate melanocytomas from melanomas by clinical signs [1], and histopathological analysis is needed [8]. Enucleation is mostly recommended when secondary ocular changes appear [4]. Consequently, the literature about choroidal melanocytoma progression over a longer period is scarce [6].

## 2. Case Report

A 3-year-old male Slovak Hound with a body weight of 31.6 kg was presented at the Veterinary Practice of the University of Hohenheim. The owners recognized a red left eye immediately after a forest walk, 3 days before the initial visit. No trauma was noticed. Systemic antibiotic treatment was initiated by the referring veterinarian (enrofloxacin, 5 mg/kg, once daily). General examination showed normal physiological parameters except an increased heart rate (116 bpm) and increased blood pressure (227/126 mmHg measured by oscillometry, petMAP graphic, Ramsey, Tampa, FL, USA), which were probably both stress-induced.

**Figure 1:**
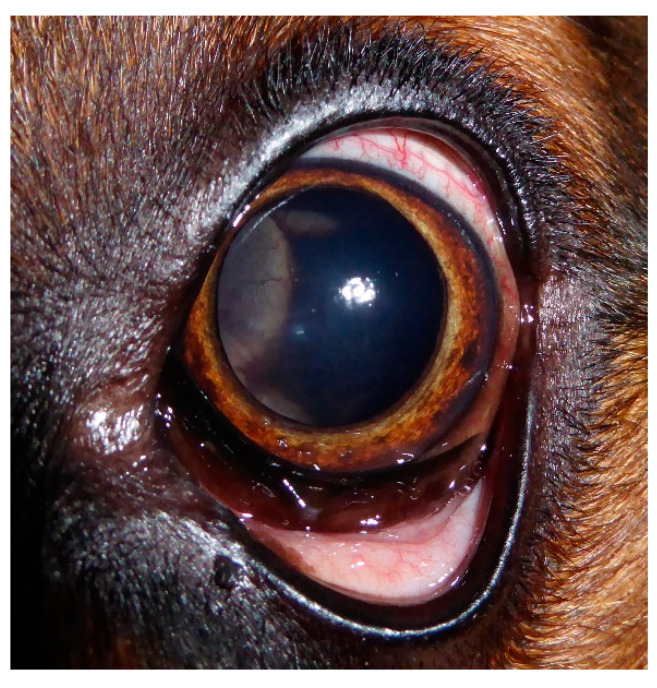
Left eye with mydriasis and episcleral hyperemia. The protrusion of the retina is visible.

Ophthalmic examination of both eyes with a portable slit lamp (SL-17, Kowa, Hamamatsu City, Japan) showed delayed pupil reaction in the right eye and absent pupil reaction in the left eye. Mydriasis and episcleral hyperemia were present in the left eye (Figure 1). The intraocular pressure (IOP) measured by tonometer (Tono-Pen XL, Mentor Ophthalmics, Norwell, MA, USA) was 13 mmHg in the right eye and 17 mmHg in the left eye. Although IOP was within physiological levels, the difference between both eyes was remarkable. With the help of an indirect binocular ophthalmoscope (Heine Omega 100, Gilching, Germany), retinal detachment was detected on the left fundus. There was a bullous protrusion of the nasal part with hemorrhagic alterations in the vitreous body. The optic disc was not visible. Retinal detachment due to trauma or infection was suspected, and anti-inflammatory eye drops (prednisolone acetate, 10 mg/mL, three times daily) and systemic cortisone treatment (prednisolone, 1 mg/kg, once daily) were prescribed.

On the next day, heart rate and blood pressure largely normalized (95 bpm, 186/93 mmHg), and IOP no longer showed any remarkable difference (left 12 mmHg and right 11 mmHg). A left pupil reaction was barely noticeable. Anti-inflammatory treatment was continued, and several blood tests were initiated.

Hematological investigations revealed a stress leukogram with neutrophilia (7.3 10^3^/mm^3^; reference range 4.8–6.6 10^3^/mm^3^), lymphopenia (0.8 10^3^/mm^3^; reference range 1.1–2.6 10^3^/mm^3^), monocytosis (0.7 10^3^/mm^3^; reference range 0–0.4 10^3^/mm^3^) and mild thrombocytopenia (193 10^3^/mm^3^; reference range 200–460 10^3^/mm^3^) (Scil Vet abc Plus, scil animal care company GmbH, Viernheim, Germany). In addition, the inflammatory marker c-reactive protein (CRP) (14.8 mg/L; reference range < 10.0 mg/L), was increased (EUROLyser Solo, Eurolyser Diagnostica GmbH, Salzburg, Austria). Blood samples were sent to the Biocontrol Veterinary Laboratory (Bioscientia Healthcare GmbH, Ingelheim, Germany) for serum protein electrophoresis, antibody titer determination and PCR tests.

Serum protein electrophoresis showed decreased alpha-1 globulin (1.6%; reference range 3.2–4.8%) and beta-1 globulin (2%; reference range 2.7–4.1%), whereas gamma globulin was increased (12.7%; reference range 6.6–12.2%). Albumin-globulin ratio was 1.4. Anaplasmosis, ehrlichiosis, toxoplasmosis and brucellosis antibody titers were all negative. *Ehrlichia canis* PCR was negative.

**Figure 2:**
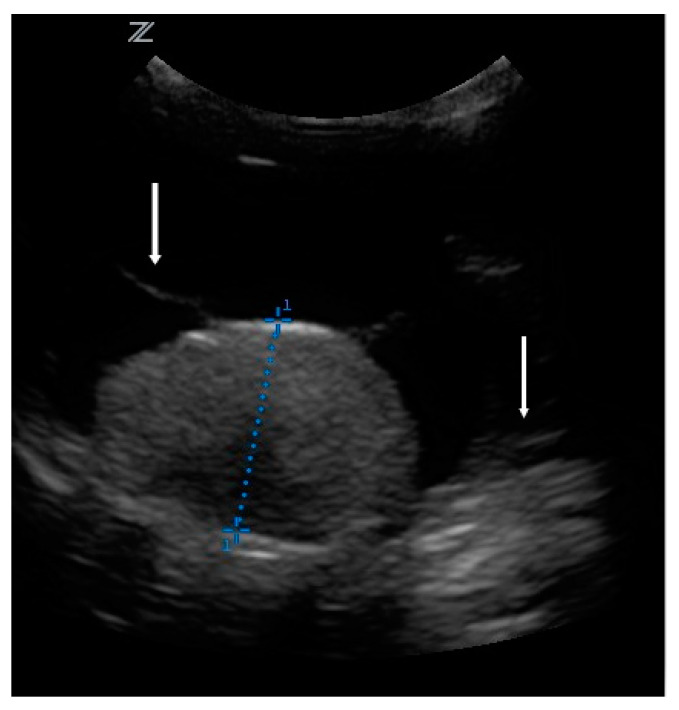
Ultrasonography of the bulbus. Diameter measured: 0.96 cm. Arrows: retina floating in the vitreous body.

The owners brought the dog in for regular check-ups every week. Although blood tests did not uncover the cause of the retinal detachment, the therapy was continued due to the clinical improvement. The condition and behavior of the dog were normal, and he did not show pain. For the next 2 months, the nasal bullous protrusion disappeared and both pupils responded to light. However, after 2 months of treatment, the protrusion of the left retina recurred, and the left pupil reaction disappeared again. This prompted further investigations. Ultrasonography (z.one ultra, Zonare medical systems GmbH, Erlangen, Germany, C 10-3 transducer, 9 MHz) of the bulbus revealed a tumor. There was a 1.5 × 0.96 cm homogeneous moderate echogenic mass on the posterior fundus. The retina was floating in the vitreous body (Figure 2). Antibiosis and systemic cortisone treatment were terminated, but local cortisone treatment continued. Enucleation and histopathological examination were recommended. The owners did not agree due to the clinical improvement. Instead, they were willing to come in for ophthalmologic and sonographic check-ups every 4 weeks for the next 3 months, and then from every 2 to 3 months.

**Figure 3:**
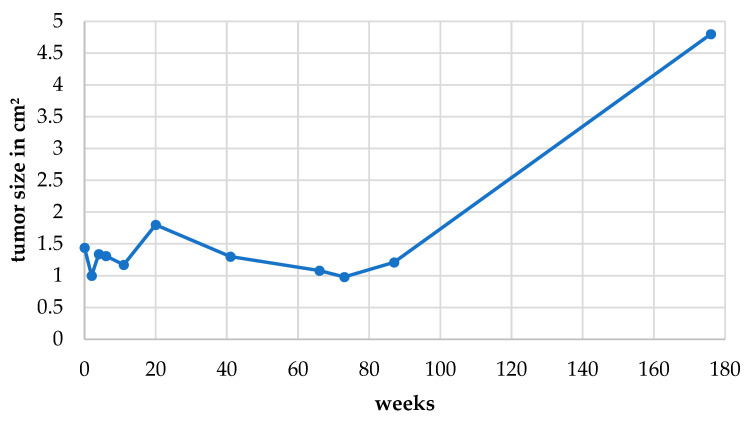
The tumor size was measured by sagittal bulbus ultrasonography, and data of length × width (cm^2^) are presented from the first to last measurement 176 weeks later.

After 1 year, a mild hyphema with low IOP (4 mmHg) was presented on consultation but disappeared upon treatment with cortisone eye drops (prednisolone acetate, 10 mg/mL, three times daily) without consequences. After 2.5 years, the secondary glaucoma occurred and caused eye irritation. The left eye showed epiphora, episcleral hyperemia and congestion. There was a small blood clot attached to the corneal endothelium, indicating intraocular hemorrhage. Secondary glaucoma was diagnosed by tonometry (IOP 22 mmHg). With treatment with brinzolamide eye drops (10 mg/mL, twice daily), the glaucoma was controlled for another year. After 3 years, the dog showed blepharospasm due to a keratitis ulcer, which was treated conventionally with antibiotic eye drops (moxifloxacin 5 mg/mL, three times daily) and vitamin A eye ointment (retinol palmitate 250 IU/g, once daily). Figure 3 shows the tumor growth over the 3-year period. For 1.5 years, the tumor did not remarkably increase in size. Alterations were within the scope of measurement accuracy. Therefore, check-ups were performed without ultrasonography. On the last examination before enucleation, the tumor was more than twice as large as on the first ultrasonography and almost filled the entire bulbus. Finally, 3 years and 5 months after the first appointment, enucleation was performed according to standard procedures.

## 3. Histopathology

**Figure 4:**
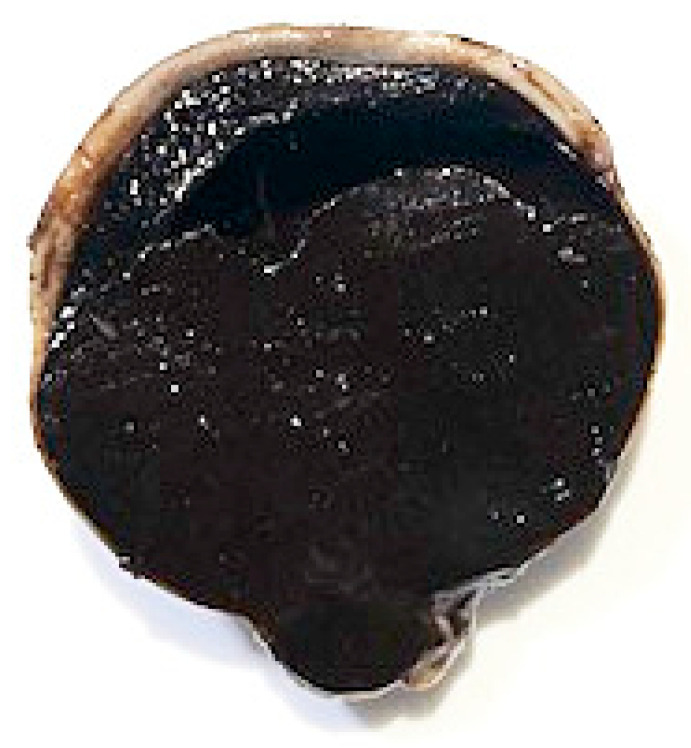
Cross-section of the bulbus. Choroidal melanocytoma with extensive infiltration of the intraocular chambers and posterior extension of the tumor in the area of the optic disc.

The bulbus, as well as three tumor bed biopsies, was fixed in 10% buffered formalin and sent to the Specialized Practice for Veterinary Pathology in Munich, Germany. Figure 4 represents the cross-section of the bulbus. The dark mass showed extensive infiltration of the intraocular chambers. Posterior extension of the tumor in the area of the optic disc was already visible macroscopically. A cross-section from the bulbus and lens and three tumor bed biopsies were paraffin-embedded, microtome-sectioned and hematoxylin-eosin-stained. Bleaching was performed to remove melanin pigment and to demonstrate cellular morphology more clearly.

**Figure 5:**
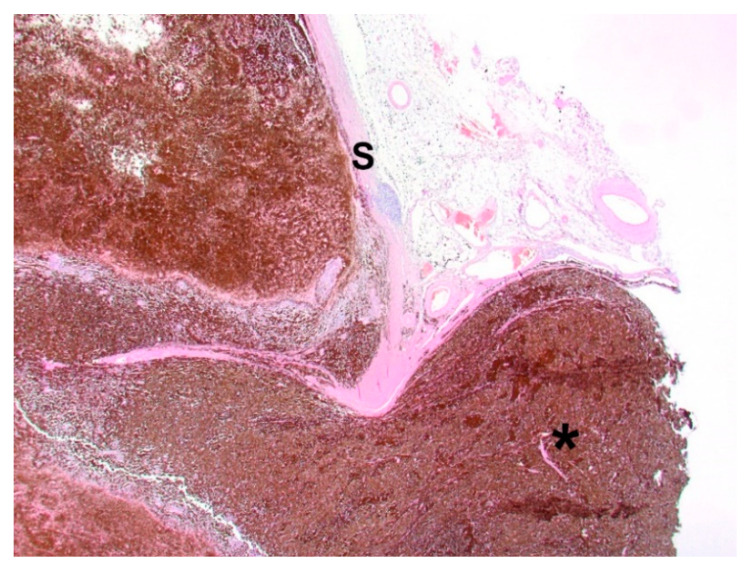
Choroidal melanocytoma with infiltration of the optic disc and extension along the optic nerve (star); S: sclera. Hematoxylin and eosin. 100× magnification.

**Figure 6:**
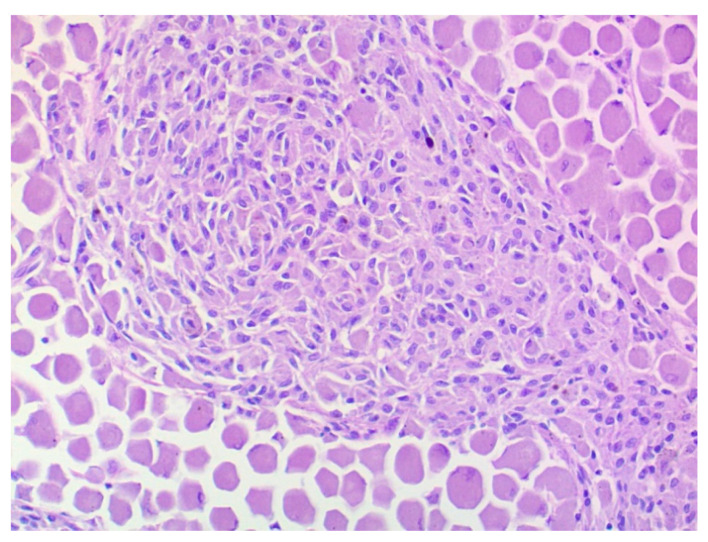
Choroidal melanocytoma: a population of large round cells with a very broad cytoplasm at the edge, and a second spindle cell population with a smaller cytoplasm in the center of the image. No significant atypia or mitoses. Bleaching, 200× magnification.

The histopathological investigation revealed a choroidal melanocytoma with partial infiltration of the sclera and optic disc, and perineural retrobulbar extension (Figure 5). The cross-section showed a diffuse infiltration with two different cell types: one plump, heavily pigmented population and a second spindle cell type of melanocytic cells. In the bleached sections (Figure 6), the two cell types showed a round to oval nucleus, respectively, and the majority of one nucleolus was visible. No significant atypia and very low mitotic activity, less than 4 mitoses in 10 high-power fields (HPF), were observed. The plump, pigmented cells could be found in the periphery of the optic nerve. No vascular invasion was found. The tumor bed biopsies confirmed retrobulbar spreading in one of the three specimens. There were pigmented cells without atypia, loosely interspersed and partly more densely packed between the skeletal musculature and the local adipose tissue.

Furthermore, extensive secondary changes were observed. Namely, a hypermature cataract and extensive posterior synechia were visible. There was a chronic, lymphoplasmacytic anterior uveitis and an extensive, pre-iridial fibrovascular membrane (PIFM) with a peripheral anterior synechia. A secondary glaucoma with a chronic exposure keratitis with neovascularization was also found.

## 4. Outcome

Enucleation was curative. At 1.5 years after surgery, no signs of tumor recurrence were found.

## 5. Discussion

Acute red eye is commonly reported by owners in veterinary practice. Redness is a nonspecific sign for inflammation [9]. We diagnosed uveitis and retinal detachment by indirect ophthalmoscopy. The patient was a young hunting dog, and the symptoms appeared after a forest walk. For that reason, anamnesis and signalement suggested infection or trauma. Hematological investigations revealed a nonspecific inflammatory condition. CRP, as an acute phase protein, and gamma globulin were lightly increased. Thrombocytopenia was not clinically relevant but could be a sign for tick-borne diseases such as anaplasmosis or ehrlichiosis [10]. Anaplasmosis, ehrlichiosis, toxoplasmosis and brucellosis can cause uveitis symptoms [9,10,11] and were all ruled out. Hunting dogs have access to prey and cadavers. Therefore, they face a higher risk of tick infestation. *Ehrlichia canis* is endemic in countries bordering the Mediterranean Sea but is spreading north to countries such as Germany [10]. The prevalence of *Anaplasma phagocytophilum* is high in Germany, and rates have been described up to nearly 50% [10,12]. Toxoplasmosis is rare in dogs, but uveitis could be the first clinical symptom [13]. IgM antibodies of *Toxoplasma gondii* are related to the acute phase of the infection. Later, IgM antibodies convert to IgG antibodies [14]. In this case, both titers were negative. *Brucella canis* is normally found in dog kennels but is also detected in wild canids and felines [15]. This could also be a source of infection. Singular titer determination is not suitable to rule out acute infections with anaplasmosis, ehrlichiosis and brucellosis. These infections should be controlled between 2 and 4 weeks for ehrlichiosis and anaplasmosis [10], and at least twice in 30-day intervals for brucellosis [15]. Serology was not repeated because of the clinical improvement and the absence of other systemic symptoms. The clinical improvement included the disappearance of the retinal protrusion and positive pupil reactions of both eyes. Consequently, we continued the anti-inflammatory treatment with cortisone eye drops, as well as the systemic antibiotic treatment that was already started by the referring veterinarian.

All infections that cause uveitis and choroiditis, respectively (fungal, bacterial or rickettsial), as well as hypertension, immune-mediated diseases and inherited conditions, can lead to retinal detachment. They can occur as a consequence of trauma, cataract surgery, luxated lenses, retinal cysts, membranes in the vitreous body, choroidal tumors or optic nerve colobomas [16]. Intraocular larva migrans has also been described to cause retinal detachment [17] but were not considered in this case. We did not expect a neoplasia in a young dog of 3 years of age. However, 2 months later, the retinal protrusion returned, and the left pupil did not respond to light any more. Ultrasonography revealed an intraocular mass.

Veterinary pathology databases show that uveal melanocytoma is the most common intraocular tumor in dogs, with a prevalence of 41.5%. Uveal melanocytoma mostly arises from the anterior uvea, with only 6% arising from the choroid, as in this case. Iridociliary adenomas with 21.1% prevalence, and uveal malignant melanomas with 13.1% prevalence, are also commonly observed. Other tumors, namely lymphomas, metastatic neoplasms, iridociliary adenocarcinomas, optic nerve meningiomas, histiocytic sarcomas, peripheral nerve sheath tumors, astrocytomas and medulloepitheliomas, are rare [1]. That means that the great majority of canine intraocular tumors are melanocytic and benign. Uveal melanocytic tumors are about 75% benign melanocytomas and 25% malignant melanomas [2,3]. Choroidal melanocytic tumors are even less malignant (15%) [1]. It is not possible to differentiate melanocytomas from melanomas by clinical signs without histopathological analyses [1,6,18], so enucleation is mostly recommended [8]. Fine needle aspiration of ocular tumors increases the risk of inflammation, infection and hemorrhage [18]. The procedure must be performed under general anesthesia and is often not diagnostic [19,20]. Case reports on magnetic resonance imaging (MRI) of canine ocular melanomas have shown characteristics similar to those described in human medicine [20,21]. This could be a future possibility to differentiate melanomas from melanocytomas. The risk of a malignant melanoma should be intensely discussed with the owners. Although uveal melanomas rarely metastasize [2,5,6], they have been described in several organs, such as the prostate [22], lungs, lymph nodes, brain, vertebra, kidneys and liver [23]. Metastasis usually occurs via the hematogenous route [18].

After 2 months of treatment and clinical improvement, the owners did not agree to enucleation. Many owners wish to keep both eyes even if they are nonvisual. Reasons could be fear of anesthesia risks, post-operative aesthetics, quality of life and impact on the human–canine bond [24].

It is considered acceptable if a patient with a choroidal melanocytic mass is monitored until the eye becomes painful or nonvisual [6,19]. The efficacy of enucleation in preventing metastasis is unproven [2]. Similarly to this case, many melanocytic tumors show transscleral extension into the orbit and optic nerve [5]. In a study by Badanes et al. [6], over 50% of patients showed evidence of tumor invasion in the optic nerve [6].

The therapies currently available for ocular melanocytic tumors are surgery, cryotherapy, radiotherapy, photodynamic therapy and laser therapy. As there are no guidelines available, the choice depends on the ophthalmologist’s preferences and owner’s financial restrictions [23]. Special expertise and equipment are needed for all therapies except enucleation. Recently, radiotherapy has emerged as the most common treatment for larger choroidal melanocytic tumors in human medicine. The transpupillary diode laser mass ablation has been described to be successful in dogs [6]; however, it is only effective for small masses that do not involve the optic nerve [4]. Chemotherapy seems to be ineffective [8]. A study of a DNA vaccination with xenogeneic human tyrosinase included one dog with an intraocular melanoma [25]. However, further studies are needed to verify the effectiveness in keeping patients in remission [19].

In the literature, as soon as secondary ocular changes appear, enucleation is recommended [4,8]. In this case, several episodes of intraocular hemorrhage appeared but were temporarily managed with cortisone eye drops. The secondary glaucoma was also treated conventionally with a carbonic anhydrase inhibitor and controlled for almost 1 year. A secondary keratitis ulcer was treated with antibiotic eye drops and vitamin A eye ointment. Enucleation was indicated as soon as bulbus destruction increased and glaucoma could not be further controlled. This occurred 3 years and 5 months after the first appointment. Histopathology confirmed a benign choroidal melanocytoma with the infiltration of the optic disc and extension along the optic nerve. The prognosis was considered good, but due to the spread along the optic nerve, the patient needed to be monitored. Enucleation was curative in this case, which is consistent with the literature [4,18,23]. At 1.5 years after surgery, no signs of tumor recurrence were found.

To our knowledge, this is the first report describing the progression of a manifest intraocular tumor with secondary complications over such a long period of time. In previous case reports, all tumors were diagnosed incidentally in visual eyes. One case report of a beagle, 13 months old, describes a minimum increase in a 2 mm small benign choroidal melanocytoma over a 7-year period without secondary ocular complications [26]. A retrospective study documented five choroidal melanocytic tumors without therapy, with no progression from 16 to 47 days [6].

## 6. Conclusions

This case history questions the standard procedure of performing enucleation as soon as clinical signs appear. The secondary complications were temporary, and the bulbus had been preserved for years with conventional treatment. Conventional treatment may be a suitable alternative to prevent anesthesia risks or if owners wish to keep the eyes. However, owners must be willing to comply with regular check-ups.

## Data Availability

The data presented in this study are available in the manuscript.
